# Train Station Pedestrian Monitoring Pilot Study Using an Artificial Intelligence Approach

**DOI:** 10.3390/s24113377

**Published:** 2024-05-24

**Authors:** Gonzalo Garcia, Sergio A. Velastin, Nicolas Lastra, Heilym Ramirez, Sebastian Seriani, Gonzalo Farias

**Affiliations:** 1 College of Engineering, Virginia Commonwealth University, Richmond, VA 23284, USA; garciaga3@vcu.edu; 2 School of Electronic Engineering and Computer Science, Queen Mary University of London, London E1 4NS, UK; 3 Department of Computer Engineering, Universidad Carlos III de Madrid, 28911 Leganés, Spain; 4 Escuela de Ingeniería Eléctrica, Pontificia Universidad Católica de Valparaíso, Valparaíso 2362804, Chile; nicolas.lastra.m@mail.pucv.cl (N.L.); heilym.ramirez@pucv.cl (H.R.); gonzalo.farias@pucv.cl (G.F.); 5 Escuela de Ingeniería de Construcción y Transporte, Pontificia Universidad Católica de Valparaíso, Valparaíso 2362804, Chile; sebastian.seriani@pucv.cl

**Keywords:** artificial intelligence, computer vision, convolutional neural networks, pedestrian detection, pedestrian tracking, human pose

## Abstract

Pedestrian monitoring in crowded areas like train stations has an important impact in the overall operation and management of those public spaces. An organized distribution of the different elements located inside a station will contribute not only to the safety of all passengers but will also allow for a more efficient process of the regular activities including entering/leaving the station, boarding/alighting from trains, and waiting. This improved distribution only comes by obtaining sufficiently accurate information on passengers’ positions, and their derivatives like speeds, densities, traffic flow. The work described here addresses this need by using an artificial intelligence approach based on computational vision and convolutional neural networks. From the available videos taken regularly at subways stations, two methods are tested. One is based on tracking each person’s bounding box from which filtered 3D kinematics are derived, including position, velocity and density. Another infers the pose and activity that a person has by analyzing its main body key points. Measurements of these quantities would enable a sensible and efficient design of inner spaces in places like railway and subway stations.

## 1. Introduction

The increasing use of rail as a mass urban transportation is making the efficiency in managing stations and their daily operations, a pressing topic. An optimal distribution of the passengers during their stay on the platforms, while they move, and the way they board and alight from the trains have the potential of reducing the required time for these actions, allowing a safer and more comfortable operation for the passengers. For high-frequency services, the capacity of the station is mainly determined by the dwell time of the trains. A longer time than necessary with the trained stopped at the station implies a longer waiting time for the passengers as well as the entire system’s capacity. This justifies the need for studying the movements of the passengers while on the platforms and their boarding and alighting times; see [1,2].

Information about the motion of the passengers and their behavior is key in the design of the layout of fixed elements located at the station and on the train, including seats for passengers, areas for organized transiting, locations of trash cans, loudspeakers, etc., to name a few. As a case in point, the Metro Santiago in Chile averages 700 million passengers per year, peaking at around 2.5 million trips per day, where the platform–train interface (PTI) is the space with the highest numbers of interactions [3]. Due to safety reasons, the PTI for reduced-mobility passengers, such as those in wheelchairs, becomes a complex space, and special measures must be taken to mitigate the chances of accidents and improve the quality of the travel experience [4].

Having reliable information on a person’s position inside the station allows the estimation of key variables such as velocity, density, and others. These quantities are useful information to help determine the overall behavior of the passengers and the way they are affected by the architectural design of the station, in terms of the distribution of objects, signage, illumination, and audible devices. Due to the high volume of video data, manually based observation at the PTI becomes practically impossible. Historically, two indirect ways to automatize this task have been developed, by monitoring the use of RFID tickets for entrance to the station and the use of cellphones inside [5].

Nowadays, technology is available for a sufficiently accurate extraction of the location of elements of interests, such as passengers, from an image or a video in a fully automatized way. Computer vision is one of the most relevant techniques in the field. This is accomplished by training an artificial intelligence algorithm based on artificial neural networks to extract the location of predefined objects belonging to different classes; see [6,7]. A popular tool of such technique is YOLOv7 [8], an open-source software that is trained to detect different categories of objects from individual images. This was supplemented with a tracking capability [9,10].

With the development of artificial intelligence, vision-based recognition of human activity has also become a field of great interest for researchers due to its significant application value. One application is face pattern recognition/extraction to infer specific activities using visible or infrared cameras [11]. Pedestrian activity recognition in a metro/train station can provide an important tool for the safe and stable operation of transportation systems. Systems are required not only to detect pedestrians but also to recognize pedestrian movements and make decisions accordingly; see [12,13,14]. Works such as [15] have shown that the use of pose estimators using skeletons to recognize the activity carried out by each pedestrian increases the performance of systems that predict activity. Typical daily activities in subway station scenarios are walking, sitting, standing, etc., but it is also important and possible to recognize activities that can trigger alarms such as a pedestrian falling, someone running, among other suspicious activities.

**Figure 1:**
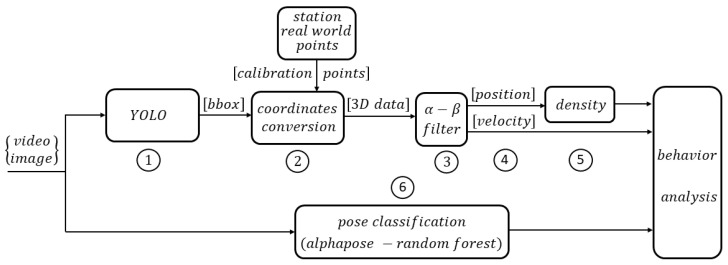
Steps carried out by the system.

The present work is aimed in that direction by proposing a pipeline that performs the following steps as seen in Figure 1: (1) Using a pre-trained YOLOv7 model complemented with the addition of tracking capability, it extracts the positions in each image of all the detectable persons in sight, delivering a bounding box framing each individual with a unique identification number; (2) it converts the positions of each person from camera coordinates to station coordinates; (3) it applies an estimation/smoothing kinematic filter to improve the passengers dynamics by smoothing their positions penalizing outliers; (4) from the kinematic filter, it obtains velocities; (5) from the position, it determines the spatial density as a function of time. Finally, the paper presents (6) a process to recognize pedestrian activity in the station, through a person’s pose classification approach.

The main contributions of this work are the following:

- Few works have been carried out on the detection and analysis of passengers by computer vision methods in metro stations (in the field of transport engineering) using the techniques described here. To the best of our knowledge, this specific application has not yet been reported in the literature.
- Obtaining transport-related variables such as speed, density, and distance, for a better assessment of the monitoring of passengers when entering/exiting the train (detection and analysis).
- A validation of the detection of passengers on the platform with respect to the video: manual/qualitative validation. The proposed approach’s results are contrasted against a manual processing of the chosen videos, labeled as ground truth.

The manuscript is organized as follows: Section 1 is an introduction. Section 2 presents the algorithms proposed for extracting the passenger’s basic data. Section 3 shows the results of the application of the proposed artificial intelligence methods to actual videos recorded inside a subway station, and a qualitative analysis from these results. The paper ends with the main conclusions in Section 4.

## 2. Materials and Methods

### 2.1. Detection and Tracking

Detection was carried out by the open-source object detector YOLOv7, augmented with the addition of a tracking package. It accepts frames from a video or images as inputs and delivers a bounding box and tracking data of each person as output. The algorithm allows for the detection of 80 different classes of objects, but in the present work, only the person and train classes were used.

#### 2.1.1. YOLOv7 (See [8])

It is a single-shot detector, comprising several convolutional neural networks in cascade trained to detect and localize objects. YOLOv7 was trained on the MS COCO dataset [16] from scratch without using other datasets or pre-trained weights. It was trained to detect some specific classes of objects, about 80 classes, and outputs for each frame all the detected objects with the vertices of a bounding box and the probability associated with a confidence level. YOLOv7 has several advantages with respect to precision, accuracy, and computational time, in comparison with its previous versions or other algorithms: it is 120% faster, allowing a range between 5 to 160 FPS; it has a faster inference speed and higher detection accuracy, which is very effective for this work; and it allows us to work in real time with lesser computational time.

#### 2.1.2. Tracking Capability Added to YOLOv7

There is also an addition to YOLOv7 allowing for tracking capability and another with segmentation, but for this work, we used the tracking version only [9,10]. This was achieved by initially assigning each detected object a unique detection number, which was kept in the following frames corresponding to the same person or train, in this case. This particular version kept the identification number stored in case an object disappeared for a few frames and then reappeared. For the frames where this occurred, no estimated positions were generated. If the gap was larger, then the algorithm assigned a new number, i.e., it considered that it was a new object.

In other words, YOLOv7 has the capability of tracking objects throughout the image or frames. This is possible due to the Simple Online and Real-time Tracking (SORT) algorithm, which allows multiple-object tracking in real time in 2D. SORT is based on simple data association and state estimation techniques. When tracking is lost for a brief period of time it does not delete the label of a bounding box. However, this algorithm does not resolve problems with occlusion or re-entering objects, it just works as a method for tracking visible objects. The key to the method is that data association (matching an object in the current frame to an object in the previous frame) is based on object appearance (e.g., as given by pixel values or an internal deep network representation).

**Figure 2:**
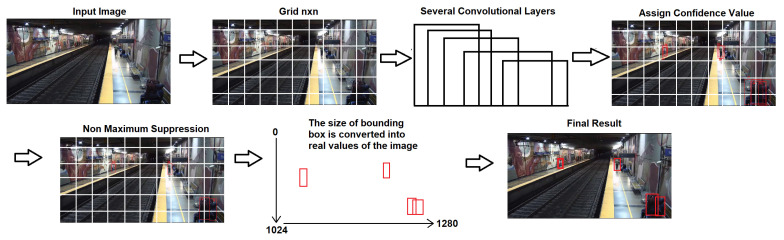
Steps carried out by YOLOv7.

YOLOv7 can be set up with different parameters when performing inference. This is a useful option for adjusting the weights, threshold, classes, augmented inferences, etc. In the near future, this work will be implemented in real time, and the YOLOv7 algorithm will be an appropriate option for detection due to its efficient use of convolutional neural networks. Detection performed with a deep learning approach is becoming faster with respect to algorithms that use regions of interest (ROIs) or multiples steps [8]. The YOLOv7 algorithm steps are: 1.- The input can be an image or video; if the input is a video it is separated into frames. Each frame is divided into cells, or grid divisions, a process that helps to better predict the object or class in the frame. 2.- Once the cells are generated, prediction is performed on each cell, generating a number of partially overlapping bounding boxes within the cells for each object. 3.- The model assigns a confidence value to each one of the bounding boxes. 4.- As the inference process generates multiple bounding boxes for the same object, a “Non Maximum Suppression” algorithm is applied, leaving the one with the highest confidence value, per object, and deleting the rest. 5.- The sizes of bounding boxes are converted into real values of the image (pixels), and then the algorithm filters the bounding boxes with a confidence threshold; see Figure 2.

### 2.2. Camera Calibration

**Figure 3:**
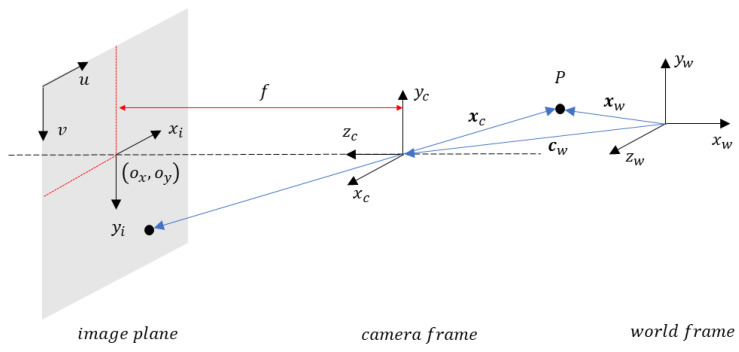
Camera calibration geometry.

One of the main steps in obtaining useful data is to convert the data from the camera’s 2D image plane (given in pixels) to data in the real world in 3D, in distance units. The process of determining the conversion parameters is called camera calibration. Consider the geometry detailed in Figure 3.

It defines the geometry relating a point in three coordinate frames: a world coordinate frame, the camera coordinate frame, and the image plane coordinate plane. The 3D transformation from world to camera coordinate frames requires the camera translation vector and orientation matrix, with respect to the world. Going from camera to image plane coordinate frames, a 3D-to-2D transformation, requires the focal length of the camera and the principal point.

#### 2.2.1. Forward Imaging Modeling

It consists in converting a point from the world coordinates to the image plane coordinates. Consider a point P located in world coordinates at the vector $x_{w}={\left[x_{w},y_{w},z_{w}\right]}^{T}$. In the camera frame, it becomes:

(1) $$x_{c}=\left[\begin{array}{c} \begin{array}{c} x_{c} \end{array}\\ y_{c}\\ z_{c} \end{array}\right]=R(x_{w}-c_{w})=\left[\begin{array}{ccc} r_{11} & r_{12} & r_{13}\\ r_{21} & r_{22} & r_{23}\\ r_{31} & r_{32} & r_{33} \end{array}\right]\left[\begin{array}{c} x_{w}\\ y_{w}\\ z_{w} \end{array}\right]+\left[\begin{array}{c} t_{x}\\ t_{y}\\ t_{y} \end{array}\right]$$

with R the rotation matrix, $c_{w}$ the position vector, and $-Rc_{w}={\left[t_{x},t_{y},t_{z}\right]}^{T}$ the translation vector of the camera with respect to the world coordinates. The two parameters $\left(R,c_{w}\right)$ represent the extrinsic camera parameters.

On the other hand, applying the perspective projection principle allows the conversion from $x_{c}$ to image coordinates. From the geometry between the point and the image plane,

(2) $$x_{i}=f\frac{x_{c}}{z_{c}},y_{i}=f\frac{y_{c}}{z_{c}}$$

with *f* the focal length. Further conversion from image plane to pixel coordinates is done by

(3) $$\begin{array}{c} u=m_{x}x_{i}+o_{x}=m_{x}f\frac{x_{c}}{z_{c}}+o_{x}=f_{x}\frac{x_{c}}{z_{c}}+o_{x}\\ v=m_{y}y_{i}+o_{y}=m_{y}f\frac{y_{c}}{z_{c}}+o_{y}=f_{y}\frac{y_{c}}{z_{c}}+o_{y} \end{array}$$

with $m_{x}$ and $m_{y}$ the pixel densities per axis, and $\left(o_{x},o_{y}\right)$ the principal point. The four parameters $\left(f_{x},f_{y},o_{x},o_{y}\right)$ represent the intrinsic parameters of the camera.

#### 2.2.2. Homogeneous Coordinates

To simplify the calculations, a linear model was derived by converting the previous equations to homogeneous coordinates as follows; see [17]. For the camera-to-image-plane conversions:

(4) $$\left[\begin{array}{c} z_{c}u\\ z_{c}v\\ z_{c} \end{array}\right]=\left[\begin{array}{c} f_{x}x_{c}+z_{c}o_{x}\\ f_{y}y_{c}+z_{c}o_{y}\\ z_{c} \end{array}\right]\left[\begin{array}{cccc} f_{x} & 0 & o_{x} & 0\\ 0 & f_{y} & o_{y} & 0\\ 0 & 0 & 0 & 1 \end{array}\right]\left[\begin{array}{c} x_{c}\\ y_{c}\\ z_{c}\\ 1 \end{array}\right]$$

and for the world-to-camera conversion:

(5) $$\left[\begin{array}{c} x_{c}\\ y_{c}\\ z_{c}\\ 1 \end{array}\right]=\left[\begin{array}{cccc} r_{11} & r_{12} & r_{13} & t_{x}\\ r_{21} & r_{22} & r_{23} & t_{y}\\ r_{31} & r_{32} & r_{33} & t_{z}\\ 0 & 0 & 0 & 1 \end{array}\right]\left[\begin{array}{c} x_{w}\\ y_{w}\\ z_{w}\\ 1 \end{array}\right]$$

and from the world to image plane

(6) $$\left[\begin{array}{c} z_{c}u\\ z_{c}v\\ z_{c} \end{array}\right]=\left[\begin{array}{c} f_{x}x_{c}+z_{c}o_{x}\\ f_{y}y_{c}+z_{c}o_{y}\\ z_{c} \end{array}\right]=P\left[\begin{array}{c} x_{w}\\ y_{w}\\ z_{w}\\ 1 \end{array}\right]$$

with the projection matrix:

(7) $$P=\left[\begin{array}{cccc} p_{11} & p_{12} & p_{13} & p_{14}\\ p_{21} & p_{22} & p_{23} & p_{24}\\ p_{31} & p_{32} & p_{33} & p_{34} \end{array}\right]$$

#### 2.2.3. Projection Matrix Estimation

The projection matrix can be estimated from a set of 3D points; see [18]. These points can be randomly chosen and should be spread out through the volumetric space of interest. Assuming i=1⋯n 3D points $\left(x_{w}^{i},y_{w}^{i},z_{w}^{i}\right)$ and their corresponding pixel coordinates $\left(u^{i},v^{i}\right)$, the following matrix equation is established:

(8) $$\left[\begin{array}{cccccccccccc} \begin{array}{c} x_{w}^{1} \end{array} & y_{w}^{1} & z_{w}^{1} & 1 & 0 & 0 & 0 & 0 & -u_{1}x_{w}^{1} & -u_{1}y_{w}^{1} & -u_{1}z_{w}^{1} & -u_{1}\\ 0 & 0 & 0 & 0 & x_{w}^{1} & y_{w}^{1} & z_{w}^{1} & 1 & -v_{1}x_{w}^{1} & -v_{1}y_{w}^{1} & -v_{1}z_{w}^{1} & -v_{1}\\ \vdots & \vdots & \vdots & \vdots & \vdots & \vdots & \vdots & \vdots & \vdots & \vdots & \vdots & \vdots \\ x_{w}^{n} & y_{w}^{n} & z_{w}^{n} & 1 & 0 & 0 & 0 & 0 & -u_{n}x_{w}^{n} & -u_{n}y_{w}^{n} & -u_{n}z_{w}^{n} & -u_{n}\\ 0 & 0 & 0 & 0 & x_{w}^{n} & y_{w}^{n} & z_{w}^{n} & 1 & -v_{n}x_{w}^{n} & -v_{n}y_{w}^{n} & -v_{n}z_{w}^{n} & -v_{n} \end{array}\right]\left[\begin{array}{c} p_{11}\\ p_{12}\\ \vdots \\ p_{33}\\ p_{34} \end{array}\right]=\left[\begin{array}{c} 0\\ 0\\ \vdots \\ 0\\ 0 \end{array}\right]$$

Equation (8), expressed in compact form, Ap = 0, can be solved by minimizing the loss function $L\left(p,\lambda \right)=p^{T}A^{T}Ap-\lambda \left(p^{T}p-1\right)$ by solving the eigenvalue problem $A^{T}Ap=\lambda p$. The vector **p** corresponds to the eigenvector associated to the smallest eigenvalue.

#### 2.2.4. Inverse Estimation

The forward imaging estimation suggests a mechanism to implement an inverse method for obtaining world coordinate points from image plane coordinates; see [19] for a similar approach. A truly 2D-to-3D transition is unambiguously achieved by the operation of two or more cameras with overlapping images. The use of a single camera imposes a limitation in the number of components to be converted. In this work, it was assumed that one of the components of a 3D point was known, i.e., the vertical component, as explained below. This assumption rendered the inverse problem feasible.

For a world point $\left(x_{w},y_{w},z_{w}\right)$, it is clear from (6) and (7), that by assuming that the component $z_{w}$ is known, together with a matrix P and its pixel coordinates (u,v), the components $x_{w}$ and $y_{w}$ are uniquely determined as follows:

(9) $$\begin{array}{c} x_{w}=g_{x}\left(P,u,v_{l},z_{w}\right)\\ y_{w}=g_{y}\left(P,u,v_{l},z_{w}\right)\\ a_{w}=h\left(P,v_{h},x_{w},y_{w}\right) \end{array}$$

**Figure 4:**
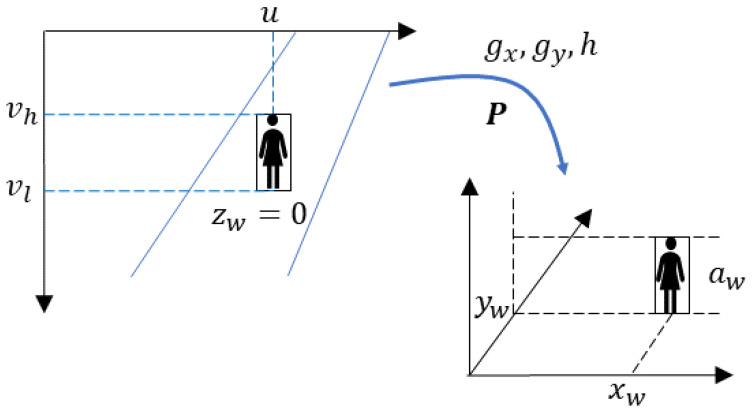
Inverse estimation.

The idea is sketched out in Figure 4. Functions $g_{x}$, $g_{y}$, and *h* carry out an inverse calculation based on (6) of the location of the person in the image plane in pixels, to the real world in world units (meters). In particular, functions $g_{x}$ and $g_{y}$, using the data set $\left\{P,u,v_{l},z_{w}\right\}$, obtain $x_{w}$ and $y_{w}$. On the other hand, *h* computes the height $a_{w}$, using the data $\left\{P,v_{h},x_{w},y_{w}\right\}$, which include the pixel coordinate of the upper edge of the bounding box $v_{h}$ and the already estimated position $\left(x_{w},y_{w}\right)$, in world coordinates.

The main objective of the present work was to determine pedestrian parameters, such as position, velocity, and spatial density, inside the train stations. Given the horizontally leveled floors of the stations under study, the assumption of a fixed and known value for the component $z_{w}$, in this case the vertical component, was reasonable and did not involve an arbitrary approximation. This was supported by the fact that in general conditions, the bounding boxes normally go from the floor up, so using a point from the bottom edge is safe as its vertical component will be practically at the floor with a known value.

### 2.3. Missing Data Estimation and Smoothing

Once the original data were converted from pixel coordinates to world coordinates, further processing was required. The method used for detection and tracking that obtains the bounding boxes per person per frame, has, from time to time, gaps for a number of consecutive frames with no information. If a person for some reason becomes not clearly visible for a short period of time, due to temporary occlusion with other people for example, the method manages to keep the same person linked before and after the gap but does not produce a bounding box.

To address this problem an estimation filter was devised. Due to the low complexity of the variables under study, i.e., position and velocity, and the lack of noise figures associated to the detection/identification process, an alpha-beta filter was used for the gap estimation. This filter also helped in smoothing the data, especially the estimated velocity. For the construction of the filter, the dynamics assumed zero acceleration, hence a constant velocity.

Given a set of points $\left(x_{w}^{j},y_{w}^{j}\right)$ associated to the same person *j*, for each frame *k*, with potential gaps in a few consecutive frames, the following prediction stage of an alpha-beta filter was constructed

(10) $$\left[\begin{array}{c} p_{\hat{x}}^{j}\left(k|k-1\right)\\ p_{\hat{y}}^{j}\left(k|k-1\right)\\ v_{\hat{x}}^{j}\left(k|k-1\right)\\ v_{\hat{y}}^{j}\left(k|k-1\right) \end{array}\right]=\left[\begin{array}{cccc} 1 & 0 & dt & 0\\ 0 & 1 & 0 & dt\\ 0 & 0 & 1 & 0\\ 0 & 0 & 0 & 1 \end{array}\right]\left[\begin{array}{c} p_{\hat{x}}^{j}\left(k-1|k-1\right)\\ p_{\hat{y}}^{j}\left(k-1|k-1\right)\\ v_{\hat{x}}^{j}\left(k-1|k-1\right)\\ v_{\hat{y}}^{j}\left(k-1|k-1\right) \end{array}\right]$$

where dt is the time interval between frames, a fixed quantity. Measured data consisted of positions $\left(p_{x},p_{y}\right)=\left(x_{w}^{j},y_{w}^{j}\right)$, and estimated/smoothed variables were position $\left(p_{\hat{x}},p_{\hat{y}}\right)$ and velocity $\left(v_{\hat{x}},v_{\hat{y}}\right)$. After a set of measurements, the update stage became

(11) $$\left[\begin{array}{c} p_{\hat{x}}^{j}\left(k|k\right)\\ p_{\hat{y}}^{j}\left(k|k\right)\\ v_{\hat{x}}^{J}\left(k|k\right)\\ v_{\hat{y}}^{J}\left(k|k\right) \end{array}\right]=\left[\begin{array}{c} p_{\hat{x}}^{j}\left(k|k-1\right)\\ p_{\hat{y}}^{J}\left(k|k-1\right)\\ v_{\hat{x}}^{J}\left(k|k-1\right)\\ v_{\hat{y}}^{J}\left(k|k-1\right) \end{array}\right]+\delta \left[\begin{array}{c} \alpha r_{\hat{x}}^{J}\left(k\right)\\ \alpha r_{\hat{y}}^{J}\left(k\right)\\ \beta r_{\hat{x}}^{J}\left(k\right)\\ \beta r_{\hat{y}}^{J}\left(k\right) \end{array}\right]$$

with $r_{\hat{x}}\left(k\right)=p_{x}\left(k\right)-p_{\hat{x}}\left(k|k-1\right)$ and $r_{\hat{y}}\left(k\right)=p_{y}\left(k\right)-p_{\hat{y}}\left(k|k-1\right)$ the a priori estimation errors, α and β the filter parameters to obtain by fine tuning, and δ={0,1} a variable to suppress the update and only allow the prediction step, when no measurements are available during the gaps in the data.

### 2.4. Ground-Truth Comparison

There are several sources of error that can potentially add up to produce biased or incorrect results. These include detection and tracking errors happening at the level of the video processing of the bounding boxes. Another source of errors can arise in the camera calibration (errors in measuring the calibration points in the field and in the extraction of their corresponding image coordinates), as this is a manual and graphical process. The latter error source can be worsened by the geometry of the station platforms, depending on the chosen camera and its orientation. When longer physical depths are involved, distant features in the image tend to get closer and be affected by the working resolution.

A preliminary assessment was carried out on the accuracy of the estimations in the two categories of errors by comparing their outcomes with a ground truth, i.e., data extracted manually from the video. First, a comparison was made between bounding boxes produced by YOLOv7 and those bounding boxes extracted manually frame by frame. Intersection over Union (IoU) gives a pertinent indicator of the goodness of the automated process, by computing the ratio of intersection and union areas between a prediction and its ground truth. Errors encountered here could include wrong classification, missed detections, and deformed bounding boxes. Secondly, the assessment was based on analyzing the resulting positions and speeds. This was achieved by contrasting the estimated values outputted by the algorithm with the values manually extracted after analyzing the video and in-site measurements. The evaluation of position accuracy was more easily performed by analyzing a person that remained in a fixed position, sitting down for example, and for speed, by choosing somebody that had a uniform motion.

### 2.5. Activity Recognition

For the recognition of activity, there are several important aspects to take into account, for example, that this is a real scenario with multiple people in the same scene, that there is occlusion with objects and occlusion between people, etc. Therefore, an activity recognition method that works in this type of scenario was necessary.

**Figure 5:**
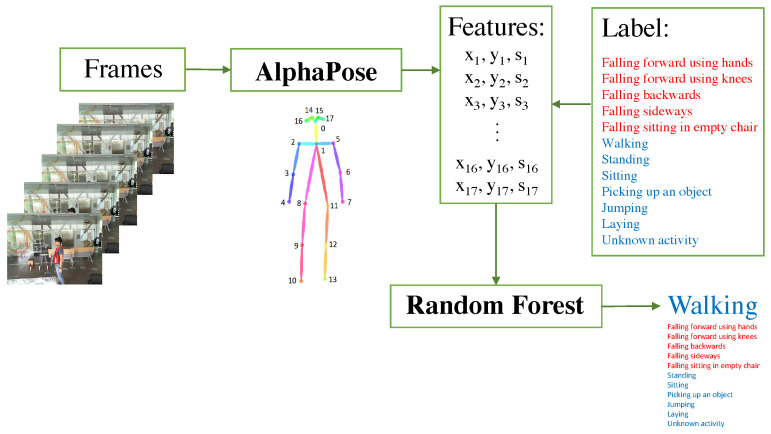
Workflow used in [15] for activity recognition.

**Figure 6:**
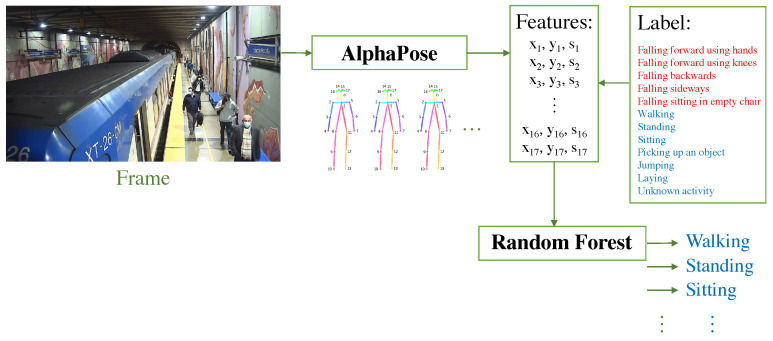
Workflow for activity recognition in this work.

It has been shown that the use of skeletons to estimate the pose of people delivers superior performance compared to other approaches for the recognition of activities, so in this work, the methodology carried out in [15] was used, as illustrated in Figure 5. The process began with the acquisition of the data (the analysis was carried out frame by frame), followed by the extraction of the poses of the people in the scene using AlphaPose [20], which generated skeletons with 17 key points (corresponding to the joints of the person’s body) each with the x and y information of the image plane plus a detection confidence score. Once the features were extracted, a feature vector was formed, so that for each skeleton, there was a vector of 51 features (16∗3) plus a label with the activity performed by the person in the scene (to carry out supervised learning). Finally, a machine learning model that predicts the activity performed by each individual was used. The workflow used for this work is shown in Figure 6, using a Random Forest (RF) model pre-trained as per the work reported in [15]. That model was trained with 12 activities, 5 falling activities (falling forward using hands, falling forward using knees, falling backward, falling sideways, and falling sitting on a flat chair) and 7 daily activities (walking, standing, sitting, picking up an object, jumping, lying down, and kneeling). In the station videos, we expected the model to mainly predict daily activities such as walking, standing, and sitting.

The main challenge was that this RF model was trained with a set of images with simulated activities, carried out by a single person in a controlled environment without any type of occlusion. However, the images of the station had multiple people, in a real uncontrolled setting and with occlusions.

## 3. Experimental Results and Preliminary Behavior Analysis

This section presents the results of the application of the algorithms detailed above to three different videos obtained from an underground train station. The process was carried out offline, but it is interesting to mention that a dedicated GPU computer would perform the same calculations in real time.

### 3.1. First Case: Limited Number of Persons

This video was selected as a proof of concept, as it contained a reduced number of persons, allowing for a simpler analysis and confirmation of the results. It had a length of 11 s, and contained five people, two of them sitting down.

**Figure 7:**
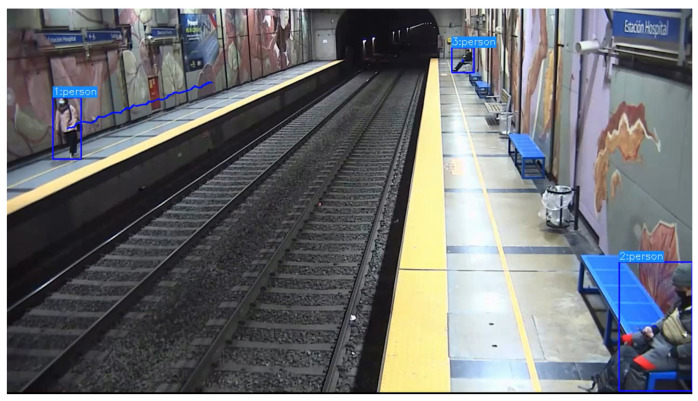
Frame example showing people in the station.

The application of the detection and tracking algorithms from YOLOv7 [8,9] generates a data set of the trajectory in pixel coordinates (u,v) with the trajectories for each detected person. Figure 7 shows a particular frame of the video with one person walking and two sitting down.

**Figure 8:**
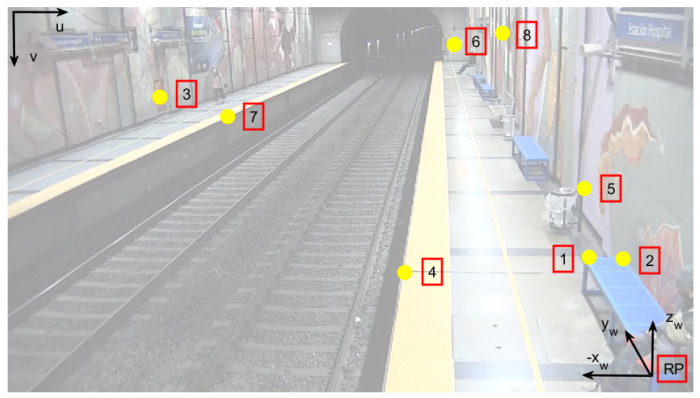
Camera snapshot of the station with points for calibration.

For the camera calibration, n=8 points were randomly selected to cover the entire volume of interest, with known world frame coordinates $\left(x_{w}^{i},y_{w}^{i},z_{w}^{i}\right)$ with respect to a chosen reference point RP, and their corresponding image coordinates $\left(u^{i},v^{i}\right)$, with i=1…n. The points used are shown on a snapshot of the camera in Figure 8 and Table 1. Points were tape-measured on site. The application of (8) gave the projection matrix *P*.

**Figure 9:**
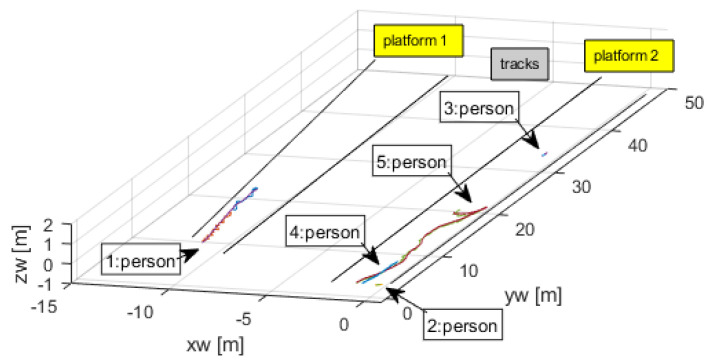
Trajectories of the tracked persons.

Having the camera calibrated, it was then possible to apply (9) to obtain the coordinates $\left(x_{w}^{i},y_{w}^{i}\right)$ for a known third component, in this case $z_{w}^{i}=-0.45$ m, which represented the vertical location of the platform floor with respect to the reference point. Figure 9 shows the world coordinate trajectories of the five persons in this video.

**Figure 10:**
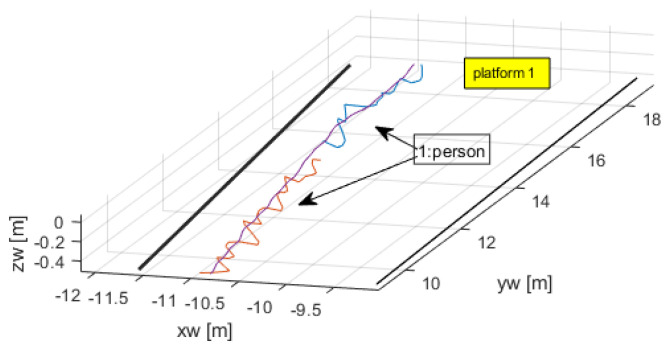
Tracking number was kept despite short absence of person.

One of the features of the open-source code for tracking and detection is that it is able to keep the correct identification of a person even if it disappears from view for a few consecutive frames. This was the case of person 1 that was absent for some frames, but whose tracking number was correctly reassigned after reappearing. This is clearly seen in Figure 10, a zoom of Figure 9.

**Figure 11:**
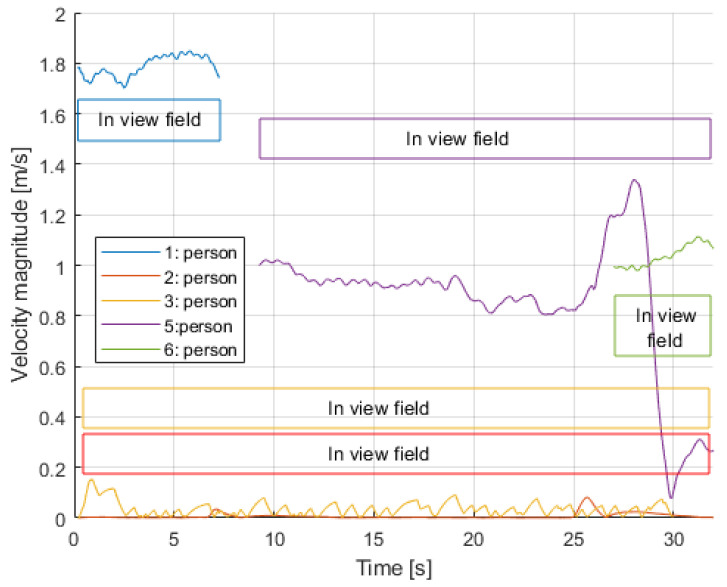
Velocity magnitude per person.

The gap in tracking is apparent from the figure. This was fixed by applying an interpolating/smoother filter, in this case an alpha-beta filter, which filled in between the two interconnected trajectories and also applied a smoothing effect for less noisy variables, specially velocity. Figure 11 shows the smoothing effect on the estimated velocities.

**Figure 12:**
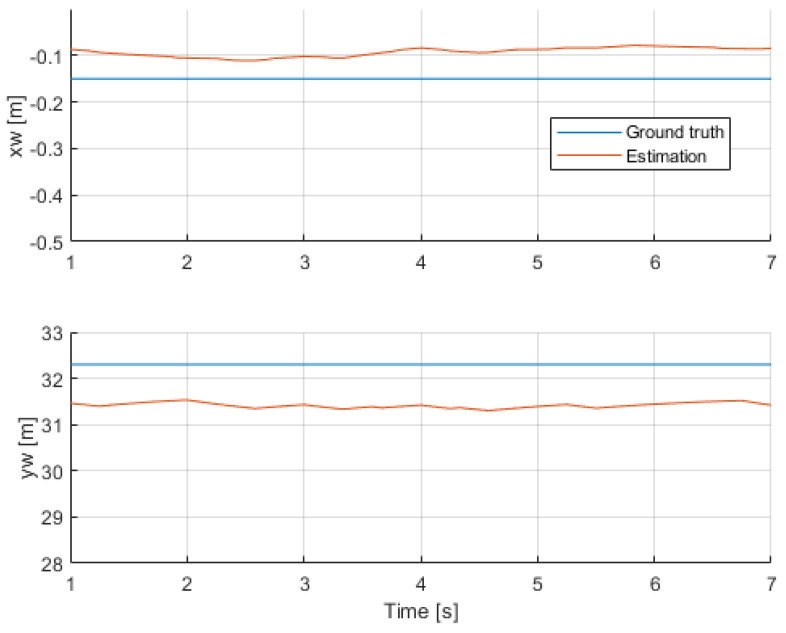
Position comparison with respect to the ground truth (person 3).

**Figure 13:**
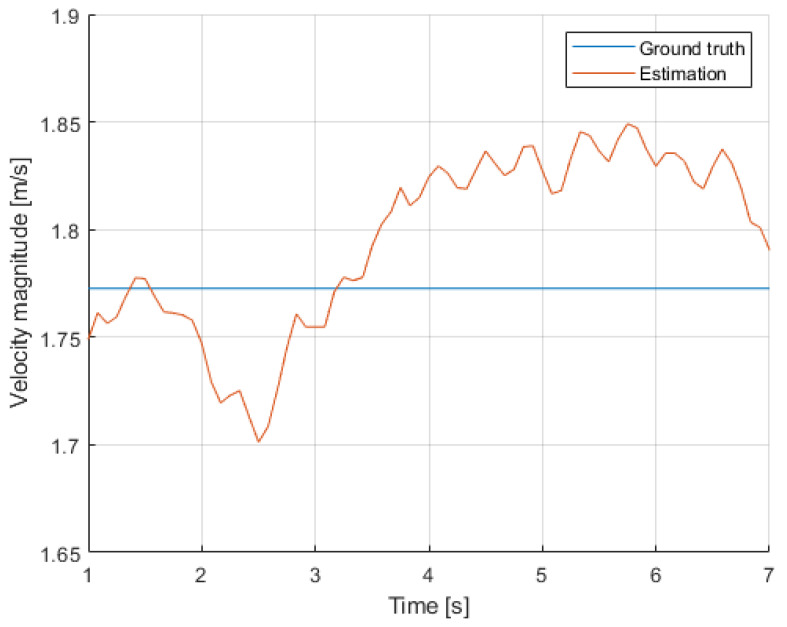
Speedcomparison with respect to the ground truth (person 1).

This video was also selected for a preliminary assessment of the accuracy of the overall estimations. It had both a person sitting down for the entire video (person 3), and a person that had a very stable motion in a portion of the video (person 1). Figure 12 and Figure 13 show the comparisons. In terms of position, component xw showed a small error around 0.1 m, and component yw had one closer to 1 meter. The estimation of speed had a better agreement with its the ground-truth value. These results served as a proof of concept and validation of the method, but further fine-tuning was required to mitigate the effect of the different sources of errors.

### 3.2. Second Case: A Larger Number of Persons

**Figure 14:**
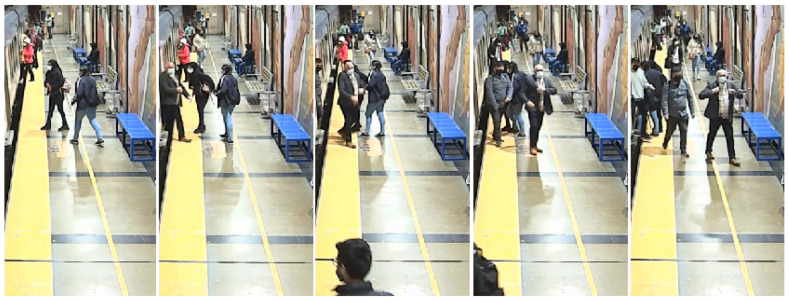
Sequence of snapshots of the second video.

As a second step, a video with a considerably larger number of persons was used. The video included the presence of a train for the entire duration of the video, so only one platform was visible. Figure 14 shows a sequence of five snapshots equally time-spaced, covering 32 persons, taken from the same camera as the first video on a different day. Camera calibration was performed with a new set of points, due to the presence of the train, so all points were chosen from the right side of the station.

**Figure 15:**
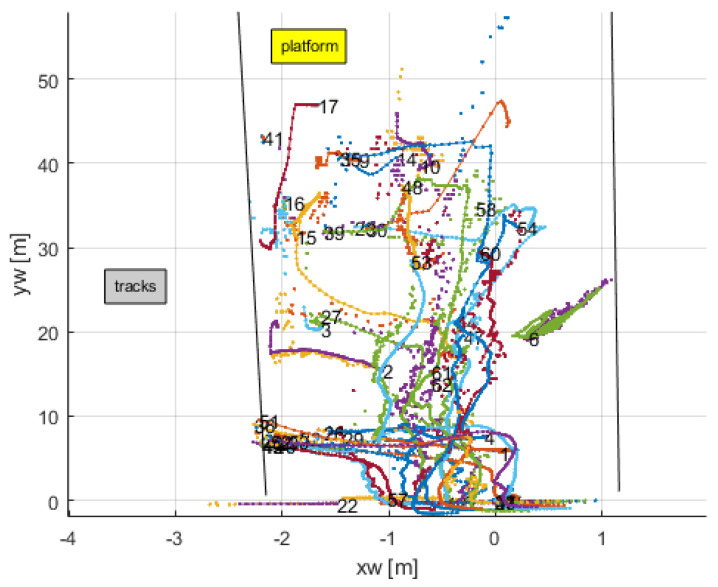
Tracked trajectories of persons.

Figure 15 shows the trajectories of the 62 persons during the video. Black lines demarcate the platform. It includes the raw data after the 2D-to-3D conversion and the filtered data. As before, the effect of the filter is clear, smoothing the motions and penalizing non-realistic outliers.

**Figure 16:**
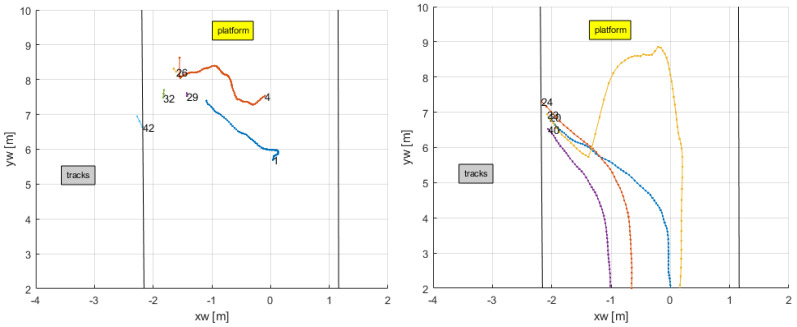
Zoom of second door in Figure 15.

**Figure 17:**
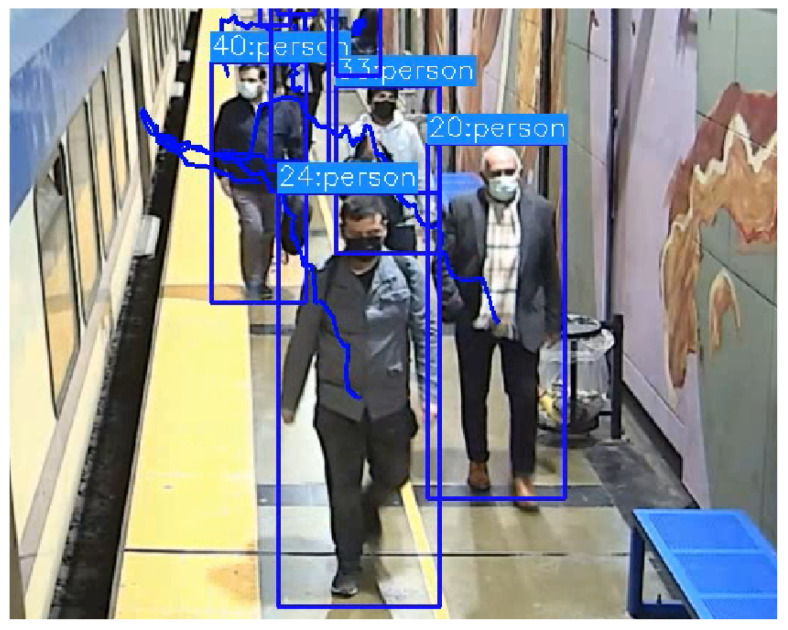
Error in bounding box of person 33.

A zoom at the location of the second door (in Figure 14 and Figure 15) shows the trajectory of some of the people as they approach to enter or leave the train, see Figure 16. Black lines demarcate the platform. Some interesting comments can be made in this case. In the upper sub-figure, it can be seen that persons 1 and 4, as they approached to board the train, suffered occlusion due to people leaving the train, and their track was lost and briefly reassigned with different identification numbers when they became visible again. On the other sub-figure, persons 20, 24, 33, and 40 left the train. Person 33 showed a departure from the trajectories the other persons were following while on the platform by walking temporarily in the opposite direction. This effect was not seen in the video, and person 33 walked along the same general path as the others. The error was due to a temporal miscalculation of its bounding box as its lower part was again occluded by other people; see Figure 17.

Here, the assumption that the lower edge of the bounding box was at a known vertical coordinate was no longer appropriate, and the conversion from 2D to 3D suffered a projection error.

**Figure 18:**
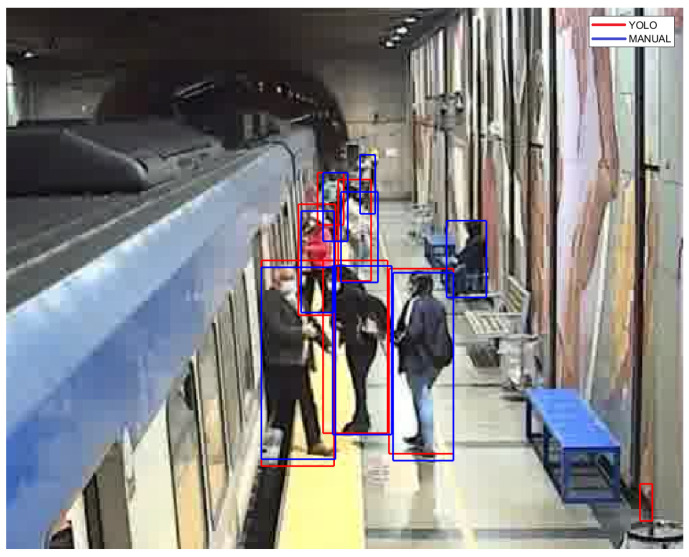
Comparison of bounding boxes.

This video was also used for an evaluation of the performance of YOLOv7. Bounding boxes generated by the method were contrasted with bounding boxes manually obtained in a subset of consecutive frames. Table 2 summarizes the average of the outcomes of the two chosen criteria, for each frame: a comparison of the number of bounding boxes correctly assigned to the class of persons is included in the first row; for correctly assigned pairs of bounding boxes, the calculation of the IoU is shown in the second row. Figure 18 zooms in on a frame with both sets of bounding boxes, where the differences can be seen more clearly.

### 3.3. Third Case: Larger-Duration Video

The final video was one of a much larger duration, around 11 min. For this reason, some trains were seen in and out of the station, and due to the orientation of the camera, in some frames, people inside the trains were detected. A total of 1081 detections were processed.

**Figure 19:**
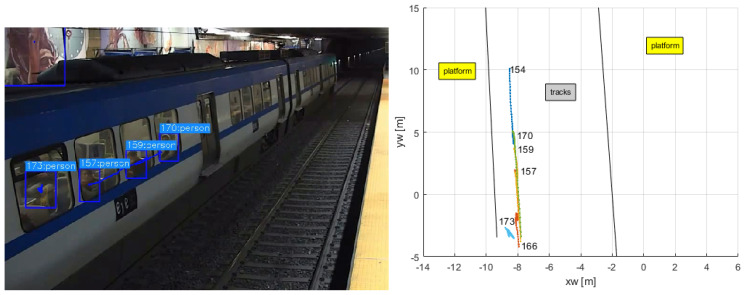
Detection of people inside the train.

The left sub-figure in Figure 19 shows a snapshot when the train was stopped at the station, and the trajectories of some people onboard the train were detected and tracked. Some of their trajectories are shown in the right sub-figure. The black lines demarcate the tracks area.

This gives the possibility of detecting people inside the tracks area when there is no train present, which would require an immediate reaction to control the situation and avoid any potential accident.

**Figure 20:**
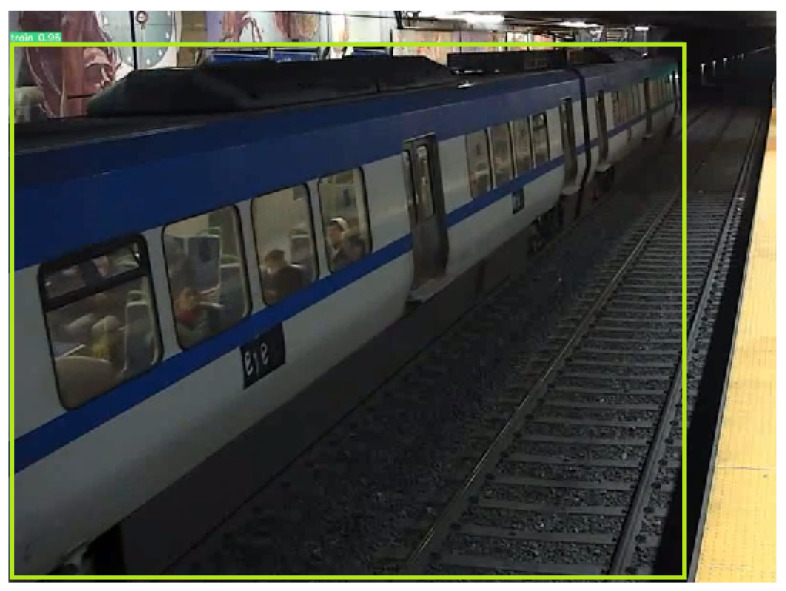
Detection of the train.

Figure 20 shows the detection of the train. As explained earlier, YOLOv7 was set to detect two classes: people and trains. The information of the train being present at the station allowed us to mask the people detected inside the train, and to focus on people detected when there is no train.

**Figure 21:**
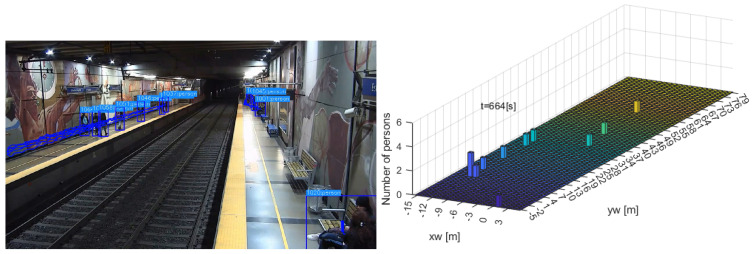
Spatial density at t=664 s, using a division of 1 square meter.

Another variable of interest was the spatial density. The analysis was conducted by subdividing the platforms into a fine grid and counted the number of persons per divisions as a function of time. Figure 21 shows the calculation of the density at t=664 s. The platform area was divided in squares of 1 square meter each. The left sub-figure shows a snapshot with the bounding boxes and part or the trajectories, and the right sub-figure shows the respective density as a number of persons sharing a division (colors do not provide extra information).

### 3.4. Activity Recognition

The last experiment consisted in recognizing the activities carried out by people in the subway station. This was achieved using the video of the first case. As mentioned in the previous section, the method consisted in passing the video frame by frame through the pre-trained Random Forest (RF) machine scanning model and predicting the activity performed by each person in the image.

Taking into account that the RF model only detected the activity of one skeleton at a time, the recognition of the activity in this work was carried out for each person in the image.

To carry out the tests, a video of the subway was used with 383 images in total, each with three or more people. All the images contained two people sitting, waiting for the subway, and at least one person walking.

**Figure 22:**
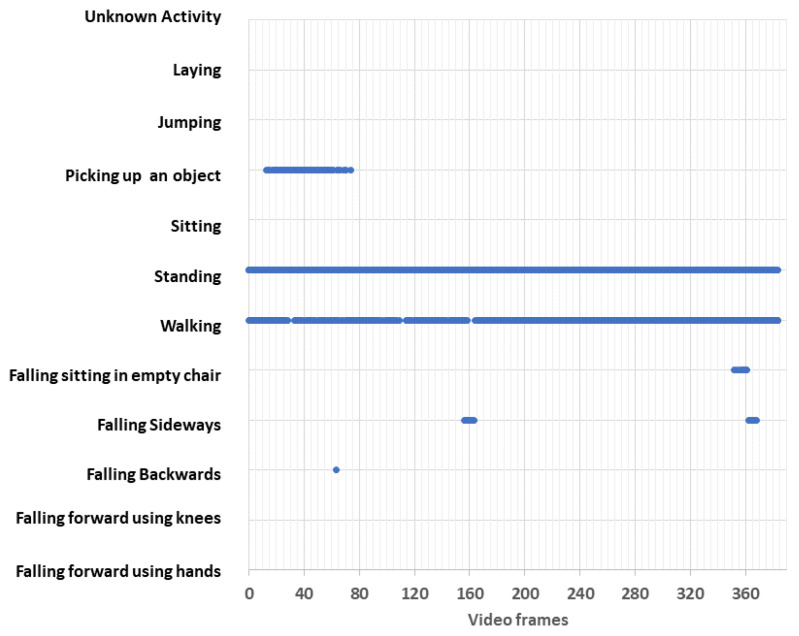
Activity recognition using the RF model.

Figure 22 shows the prediction made by the RF model. The graph shows the activity performed by people in each frame of the video. As can be seen, the model incorrectly predicted that in some images a person fell, and that in some images, a person was holding an object. Nevertheless, the RF model was able to correctly predict that there were one or more people sitting in all the images, and it was also able to predict correctly that in most of the images, there were one or more people walking.

Finally, the model incorrectly predicted three different types of falls in a total of 24 images and wrongly predicted that in 54 images, a person picked up an object. These figures are shown in Table 3.

Although the approach delivered a total of 78 “False Alarms”, the model was quite accurate at recognizing people’s activity when they were walking. It might be possible to increase the performance of the proposed model and reduce the false alarms by re-training the model with other datasets in uncontrolled, real, and occluded environments, including different camera orientations and positions, or by using other types of machine learning or deep learning models.

### 3.5. Behavioral Analysis

#### 3.5.1. Combined Kinematics and Activity Approach

From the presented results, some preliminary analysis can be conducted regarding the behavior of passengers during their presence at the station. Both methods used, i.e., the extraction of kinematic information and the classification of the specific activity, are seen as complementary, enhancing the overall analysis. Using the video of the first case, analyzing the velocities of all passengers throughout the duration of the video, see Figure 11, we can infer the following classification in Table 4:

This result complements the classification shown in Table 3, indicating that there were people sitting down the entire video (persons 2 and 3), and that there were people walking (persons 1, 5, and 6) in different but overlapping sections of the video, adding up to a value close to the one estimated in Table 3.

#### 3.5.2. Kinematics Approach

Part of the video from the third case was used for this analysis (the first 308 s). Four stages were singled out, choosing representative persons for each one. Their estimated behavior, including walking, standing or barely moving, boarding or leaving the train, can be inferred from a combination of their speeds and their estimated trajectories.

**Figure 23:**
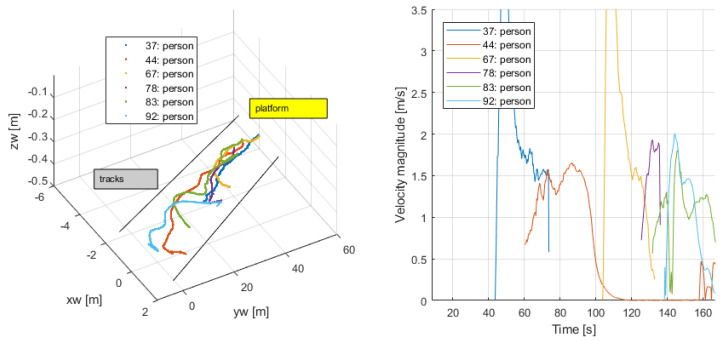
Entering the platform.

**Figure 24:**
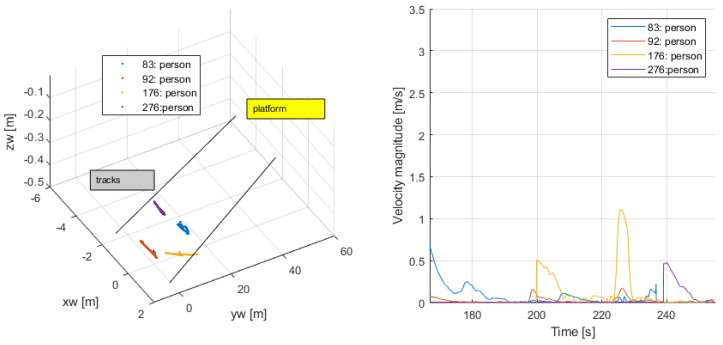
Waiting for the train.

**Figure 25:**
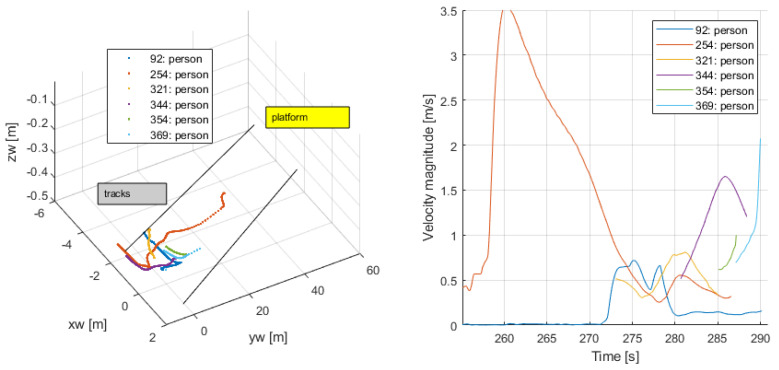
Train approaching, boarding and alighting from the train.

**Figure 26:**
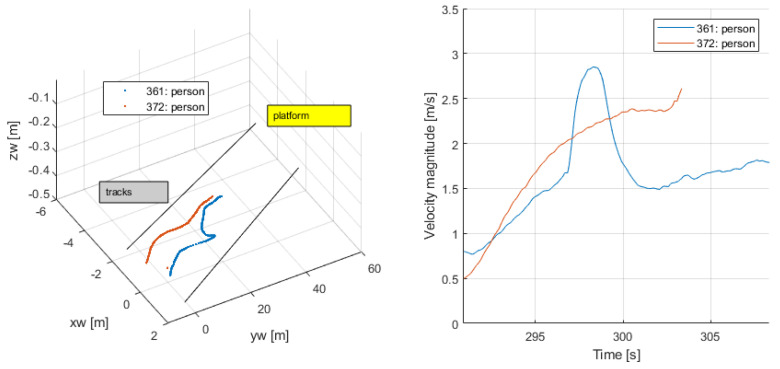
Leaving the platform.

- Walking onto the platform (0−160 s): Persons {37,44,67,78,83,92} entered the platform and reached a point and mainly stopped. Figure 23 shows their tracking history and speeds, from where this activity can be inferred. Their average speed was different from zero, indicating their motion until they settled in a fixed position.

- Waiting for the train arrival (161–255 s): Persons {83,92,176,276}, some of which may have entered the platform before the beginning of the video, remained with almost no motion waiting for the train, or briefly changed position; see Figure 24. Their average speed was close to zero, and their trajectories were closely centered at a fixed position.

- Train approaching, boarding and alighting (256–290 s): Persons {92,254,321,344} approached and entered the train through two different doors. Persons {354,369} left the train; see Figure 25. Tracking for these last ones was available until they became visible. Their speeds clearly indicated their activity, as they changed abruptly from a value different from zero, either because the passengers appeared or disappeared from one frame to the next one.

- Leaving the platform (291–308 s): Persons 361,372, after alighting from the train (from a door not in the visual field), walked out from the platform; see Figure 26. Their speed was consistent as they increased from lower values to higher ones reflecting their more stable motion towards the exit.

**Figure 27:**
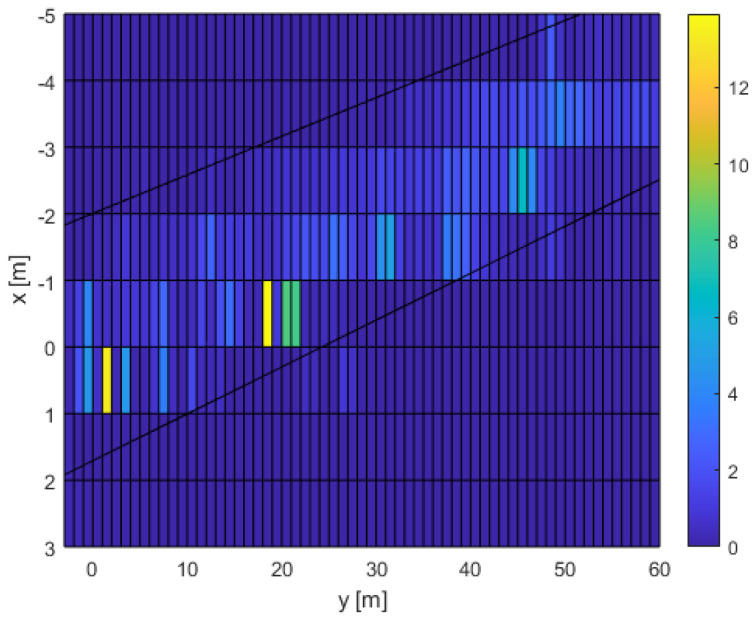
Average number of people per square meter.

Figure 27 shows the average number of people per square meter on the platform, as people circulated.

## 4. Conclusions

Knowledge of people’s behavior in train stations is critical for any optimization of space design and services to be carried out. The article aimed to test the concept of extracting kinematic and pose information of passengers from cameras on a subway platform as they entered/exited trains and as they moved around. Two complementary methods were tested, both implemented on convolutional neural networks. One was based on a kinematic analysis, from where variables such as position, velocity, and spatial density were calculated; and the other used extracted key points from the body, from which specific attitudes or poses were estimated, especially walking. The results were promising in the sense that fusing both methods, it was possible to quantitatively and qualitatively extract information useful for transport management. The approach can be further improved, making it more robust, especially the activity recognition method, by performing a fine-tuning of the neural network models with new conditions, unforeseen situations, such as different stations, different cameras and their position and orientation, and changing light levels. As a proof of concept, the present work reached its objectives to create an accessible automatized technique with standard computational resources, delivering useful raw information for an in-depth analysis towards a better station design.

## Figures and Tables

**Table 1:** Calibration points.

Calibration	Component	Component	Component
**Points**	xw **[m]**	yw **[m]**	zw **[m]**
1	0.00	2.45	0.00
2	0.45	2.45	0.00
3	−12.19	17.35	0.31
4	−2.10	3.00	−0.45
5	0.43	4.55	0.25
6	−0.62	47.30	1.05
7	−9.40	17.35	−0.45
8	0.30	17.44	2.12

**Table 2:** YOLOv7 performance.

Criterion	Outcome (%)
Number of correct bounding boxes	81.5
IoU	77.6

**Table 3:** Number of frames containing each activity.

	Total Frames	
	**Original Video**	**RF Model**
Falling forward using hands	0	0
Falling forward using knees	0	0
Falling backwards	0	1
Falling sideways	0	15
Falling sitting in empty chair	0	8
Walking	383	361
Standing	0	0
Picking up an object	0	54
Jumping	0	0
Laying	0	0
Kneeling	0	0

**Table 4:** Inferred classification from the estimated velocities.

	Total Frames				
Person	1	2	3	5	6
Walking	85	0	0	271	60
Standing	0	381	381	0	

## Data Availability

Data is contained within the article.
